# Putative roles of purinergic signaling in human immunodeficiency virus-1 infection

**DOI:** 10.1186/1745-6150-9-21

**Published:** 2014-10-29

**Authors:** Paulo AF Pacheco, Robson X Faria, Leonardo GB Ferreira, Izabel CNP Paixão

**Affiliations:** 1 Laboratory of Cellular Communication, Oswaldo Cruz Foundation, Av. Brazil, 4365 Rio de Janeiro, Brazil; 2 Laboratory of Molecular Virology (Center for Bioproducts), Universidade Federal Fluminense, Rua Outeiro de São João Batista, s/n, Campus do Valonguinho, Niterói, Rio de Janeiro, Brazil

**Keywords:** P2X7R, HIV-1 infection, Antagonists, Pharmacological targets

## Abstract

**Reviewers:**

This article was reviewed by Neil S. Greenspan and Rachel Gerstein.

Nucleotides and nucleosides act as potent extracellular messengers via the activation of the family of cell-surface receptors termed purinergic receptors. These receptors are categorized into P1 and P2 receptors (P2Rs). P2Rs are further classified into two distinct families, P2X receptors (P2XRs) and P2Y receptors (P2YRs). These receptors display broad tissue distribution throughout the body and are involved in several biological events. Immune cells express various P2Rs, and purinergic signaling mechanisms have been shown to play key roles in the regulation of many aspects of immune responses. Researchers have elucidated the involvement of these receptors in the host response to infections. The evidences indicate a dual function of these receptors, depending on the microorganism and the cellular model involved. Three recent reports have examined the relationship between the level of extracellular ATP, the mechanisms underlying purinergic receptors participating in the infection mechanism of HIV-1 in the cell. Although preliminary, these results indicate that purinergic receptors are putative pharmacological targets that should be further explored in future studies.

## Introduction

Nucleotides and nucleosides are fundamental molecules in cell metabolism that perform a wide range of acknowledged functions that include acting as energy sources and being the structural building blocks of nucleic acids [1,2]. Furthermore, these compounds act as potent extracellular signaling molecules and neurotransmitters via the activation of a family of cell-surface receptors termed purinergic receptors [3]. Based on biochemical, pharmacological and molecular biological studies, purinergic receptors are categorized into P1 receptors (for adenosine) and P2 receptors (P2Rs, for ATP/ADP and some pyrimidines) [4-8].

To date, four P1 receptors have been identified: A_1_, A_2A_, A_2B_ and A_3_. All of these molecules are typical G protein-coupled receptors, although they differ with respect to the G protein to which they are coupled [8,9]. The P2Rs are separated into two distinct families: the ionotropic P2X receptors (P2XRs) (activated by ATP) and the metabotropic P2Y receptors (P2YRs) (that bind to ATP, UTP or their metabolites) [10-12]. Mammals express seven subtypes of P2XR monomers (P2X1-7R) and eight subtypes of P2YR monomers (P2Y1R, P2Y2R, P2Y4R, P2Y6R, P2Y11R, P2Y12R, P2Y13R, and P2Y14R) [13,14].

The P2XRs function as ATP-gated ion channels. Following the binding of an agonist to their extracellular portions, they undergo conformational changes that result in the opening of an ion channel, facilitating the influx/efflux of cations [15-17]. Following prolonged activation by ATP, some P2XRs, such as P2X2R, P2X4R and P2X7R, are capable of undergoing an additional conformational change, which increases the permeability of large molecules [18-20]. In response to prolonged exposure to ATP, other P2XRs, such as P2X1R and P2X3R, undergo a fast desensitization, resulting in channel closure [17,21-23]. Functional P2XRs are assembled into either homomeric or heteromeric trimers, each subunit of which contains two transmembrane domains; a large extracellular loop, which includes 10 conserved cysteines and glycosylation sites; and intracellular N and C terminal domains, which contain consensus phosphorylation sites for protein kinases [5,6,24-26]. The detailed signaling process that is triggered by the activation of most P2XRs has yet to be completely elucidated. For instance, one possible intermediate in the activation of MAPKs, PKC and calmodulin may be the cytoplasmic calcium [25,27].

The P2YR subtypes are typical G protein-coupled receptors (GPCRs), which typically consist of seven transmembrane domains connected by three extracellular and three intracellular loops. The N-terminus is extracellular, and the C-terminus is intracellular [28-30]. Similar to other GPCRs, stimulation of P2YRs induces the activation of a heterotrimeric G protein and its dissociation into α and βγ subunits, activating a range of effector proteins, such as phospholipase C and adenylyl cyclase [25]. The P2YR family displays high diversity with respect to amino-acid composition and structural diversity with respect to the intracellular loops and the C-terminus, influencing the degree of coupling with functionally distinct types of G proteins [31,32].

P2Rs are broadly distributed throughout nervous, endocrine, cardiovascular, renal, gastrointestinal and immune tissues [33] and participate in several biological events, such as platelet aggregation, exocrine and endocrine secretion, endothelial-mediated vasodilatation, nociceptive mechanosensory transduction, neuromodulation and neuroprotection, cell proliferation, differentiation, migration and death during embryological development, wound healing, epithelial cell turnover and immune responses [14,16].

## Review

### Importance of purinergic signaling in immune responses

Most immune cells express both P1 and P2 receptor subtypes [34-39]. A large body of evidence has demonstrated that extracellular nucleotides and purinergic receptors are key regulators of many immune phenomena, such as inflammatory pain, cytokine secretion, chemotaxis, activation of various types of immune system cells, surface-antigen shedding and pathogen killing.

ATP, adenosine and other nucleotides are also involved in regulating the migratory responses of neutrophils, macrophages and other innate immune cells [40,41]. Metabolic stress, ischemia, hypoxia, inflammation and trauma lead to the accumulation of adenosine in the extracellular space, reporting tissue injury to the surrounding tissue in an autocrine and paracrine manner. Another possibility is that adenosine generates tissue-protective responses [42-44]. In its protective function, adenosine downregulates the expression of adhesion molecules, the production of oxygen radicals, degranulation and the release of cytokines, consequently reducing cellular cytotoxic activities [45-52]. In particular, ATP and UTP that have been released by apoptotic cells act as “find-me signals” that induce phagocyte migration toward these cells [53]. In neutrophils, stimulation with the chemotactic peptide *N*-formyl-Met-Leu-Phe causes a rapid release of ATP in a polarized manner. This released ATP activates P2Y2R to modulate cellular orientation. The adenosine formed via ATP degradation and subsequent stimulation of the A_3_ receptor enables autocrine signal amplification, which facilitates chemotaxis by regulating the speed of migration [54]. Similar results have been observed in macrophages, in which stimulation with the chemoattractant C5a induced migration via the release of ATP and the activities of the autocrine purinergic signaling system [55]. In addition, some studies have suggested that ATP acts as a signal that induces the release of chemotactic factors [56]. In contrast, other studies have found evidence that nucleotides themselves function as chemotactic signals [53,57,58]. Extracellular nucleotides are also involved in modulating lymphocyte responses. B cells express various P2R subtypes [38,59] and are able to release ATP under steady-state conditions [60]. Incubating human B cells with increased ATP concentration triggers a dose-dependent increase in the level of inositol 1,4,5-trisphosphate as well as an increase in the level of cytosolic free Ca^2+^. In addition, the c-fos and c-myc mRNA levels increase, indicating that P2-receptor stimulation was associated with this activity [61]. A transfection study using two P2X7R non-expressing human lymphoid cell lines (K562 and LG14 cells) showed that the heterologous expression of this receptor enhanced cell proliferation in the absence of growth factors and was dependent on the level of released ATP [62].

Similarly, several stages of the life of a T lymphocyte may be affected by purinergic signaling, including differentiation, activation and proliferation. Thymocytes are susceptible to apoptosis in the presence of extracellular ATP via the activation of purinergic receptors in vitro and in vivo [63-69]. The sensitivity of this cell type to extracellular ATP is closely associated with its degree of maturation, highlighting the importance of the purinergic system in the regulation of cellular differentiation in the thymus [70]. Recently, signaling via purinergic receptors has been associated with fate determination during T-cell development. P2X7R activation contributes to the strength of the γδTCR signal in immature thymocytes. The genetic ablation or pharmacological inhibition of this receptor directs cells toward the αβ fate [71]. Peripheral T lymphocytes release ATP into the extracellular milieu under several conditions, such as TCR stimulation, mechanical stimulation or osmotic stress [72-76], via either the pannexin 1 hemichannel [77,78] or vesicular exocytosis [79]. In these cells, the binding of extracellular ATP to P2Rs modulates several steps required for complete T-cell activation, such as the influx of extracellular calcium, the activation of p38 MAPK and the secretion of IL-2 [72,75,80-82]. In addition to conventional αβ T lymphocytes, the activation state of γδ T cells is also regulated by ATP release and autocrine signaling via a purinergic receptor, namely P2X4R [83]. Similarly, activation of P2X7R by ATP inhibits the suppressive function and disrupts the gene transcription profile of Tregs and promotes the differentiation of these cells into proinflammatory TH17 effector cells [84].

Furthermore, purinergic signaling, particularly that involving P2X7R, also acts as a potent mediator of the release of proinflammatory cytokines. One of the best studied examples of this correlation is the participation of P2X7R in the processing and release of IL-1β and IL18 [85,86]. Based on experiments performed in vitro and in vivo using P2X7^-/-^ mice, ATP was identified as a strong IL-1β-releasing agent that acts via this receptor. LPS, the strongest inducer of IL-1 secretion, is an incomplete stimulus in the absence of ATP, leading to the accumulation of pro-Il-1β in cytoplasmic vesicles [85,87].

These findings reinforce the importance of purinergic signaling mechanisms as key regulators of many aspects of immune responses. Under normal conditions, extracellular ATP (the natural agonist of most P2Rs) is present at nanomolar concentrations, which are maintained by extracellular nucleotide-hydrolyzing enzymes, such as ecto-nucleoside triphosphate diphosphohydrolases (E-NTPDases) [88-90]. However, under certain conditions, such as inflammatory, ischemic and hypoxic states, several different cell types release ATP from intracellular storage compartments into the extracellular media, elevating its external concentration to the millimolar range and inducing predominantly proinflammatory responses [90,91]. Several mechanisms for ATP release have been described, including cell death, vesicular transport, and the activities of stretch-activated channels, volume-regulated channels, maxi-anion channels, pannexin and connexin hemichannels and P2X7R [9,13,92]. In a “stressful environment” containing damaged host cells and the leakage of intracellular contents, purinergic receptors allow immune cells to recognize the ATP that is released into the extracellular milieu. Therefore, purinergic signaling participates in an important system that recognizes “danger” signals, alerting the immune system to a threat [93,94]. Accordingly, the damage caused by pathogens at the infection site induces the release of additional ATP [95].

In this regard, it appears that a natural consequence of stress conditions is the alteration of purinergic signaling, which may be related to pathological processes associated with infectious conditions.

### Involvement of purinergic receptors in HIV infection

Accordingly, some studies have investigated the involvement of purinergic receptors, particularly P2X7R, in infectious processes [96], as shown in Table 1.

**Table 1:** Involvement of purinergic in infectious processes

**Microorganism**	**Receptor**	**Cell type**	**Involvement**	**Reference**
**Bacteria**				
*M. tuberculosis*	ND	monocyte	ATP induced apoptosis of infected monocyte and reduced viability of intracellular bacilli	[97]
BCG	P2X7	Human macrophage	Treatment with exogenous ATP caused cell death and killing of intracellular mycobacteria within BCG infected macrophages	[98]
*M. tuberculosis*	P2X7	Human macrophage	Treatment with ATP reduced viability of three virulent strains of mycobacteria within human macrophages, what was associated with stimulation of phospholipase D activity	[99]
BCG	P2X7 and P2Y	Human macrophage	Apoptosis of infected cells and killing of intracellular bacilli	[100]
*M.bovis*	P2X7	Bovine macrophage	ATP induced killing Mycobacterium bovis in bovine macrophages in a mechanism P2X7R-dependent	[101]
BCG	P2X7	Murine bone-marrow derived macrophages and murine macrophage cell line	P2X7R stimulation with ATP induced rapid fusion of BCG-containing phagosomes with lysosomes, resulting in formation of multibacillary vacuoles. Also, P2X7R resulted in progressive acidification of BCG-containing phagosomes in infected macrophages	[102]
BCG	P2X7	Human macrophage	Loss-of-function polymorphism 1513A → C abolished apoptosis of infected macrophages and mycobacterial killing	[103]
BCG	P2X7	Human macrophage	The 1513C allele was associated to increased susceptibility to extracellular TB and ATP-mediated killing of mycobacteria in macrophages was absent in homozygous subjects and impaired in heterozygous subjects	[104]
BCG	P2X7	Human macrophage	Loss-of-function polymorphism 1096C → G (change Thr(357) to Ser (T357S)) associated to reduced or near to absent ATP-induced killing of intracellular mycobacteria	[105]
*M. tuberculosis*	P2X7	Human PBMC	PBMC from TB patients presented different pattern of gene expression in response to ATP when compared to healthy contacts	[106]
*M. tuberculosis*	P2X7	Human monocyte/macrophages	Mycobacterial infection induced an increase of P2X7 expression, higher release of ATP and an increment of intracellular ATP accumulation	[107]
BCG	P2X7	THP-1 and monocyte-derived macrophage	ATP treatment activated autophagy pathway via a Ca2 + -dependent process. This effect was associated with a phago-lysosomal fusion and of mycobacteria-containing phagosomes, resulting in reduction in intracellular BCG viability	[108]
*C. psittaci*	P2X7	Murine macrophage cell line	ATP but no other nucleotides was able to induce reduction in viability of intracellular bacteria and chlamydial infection prevented ATP-mediated apoptosis	[109]
*C. trachomatis*	P2X7	Murine peritoneal macrophage cells and macrophage cell line	Chlamydial killing upon ATP treatment of infected cells required phospholipase D activation, which is mediated by P2X7R stimulation that leads to lysosome fusion with mature Chlamydia vacuoles	[110]
*C. muridarum,*	P2X7	Human cervical adenocarcinoma cell line	Extracellular ATP or other P2X7R agonists induced a decrease in chlamydial viability in epithelial cells, which was dependent on phospholipase D activity and blocked by treatment with P2X7R antagonists and butan-1-ol (PLD inhibitor). Also, vaginal infection was more efficient in P2X7R-deficient mice, what was correlated to higher level of acute inflammation	[111]
**Protozoan**				
*L. amazonensis*	P2X7	Murine macrophage cell line	Native and recombinant *Leishmania* nucleoside diphosphate kinase (NdK) prevented ATP-induced cell death	[112]
*L. amazonensis*	P2X7	Murine peritoneal macrophage	*Leishmania* infection leads to increased expression of P2X7R and higher responsiveness to ATP treatment. Also, incubation with ATP reduced the parasite load, which was reverted by pre-treatment with oxidized ATP and was not not dependent of cell lysis or NO production	[113]
*L. amazonensis*	P2X7	Murine peritoneal macrophage	Macrophages infected with L. amazonensis exhibit higher apoptosis rate and parasite degradation upon ATP treatment and presented differential modulation of the uptake of cationic and anionic dyes	[114]
*L. amazonensis*	P2Y	Murine peritoneal macrophage	Uridine nucleotides reduced parasite load and induced morphological damage of intracellular parasites and infected cells. They also induced significant levels of apoptosis, ROI and RNI in infected cells.	[115]
*T. gondii*	P2X7	Murine peritoneal cell and macrophage cell line	ATP or BzATP treatment reduced parasite load. Parasite load was not reduced in P2X7R-deficient mouse. Furthermore, ATP treatment caused ultrastructural changes in tachyzoite inside macrophages, increased lysosome fusion with parasitophorous vacuole and ROS production	[116]
*T. gondii*	P2X7	Human macrophage and murine bone marrow-derived macrophage and macrophage-like cell line	Infected macrophages obtained from homozygous individuals for loss-of-function polymorphism 1513A → C had no significant alteration in parasite load after ATP treatment. Similarly, macrophages from P2X7R knockout mice were not able to kill T. gondii upon ATP treatment	[117]
*T. gondii*	P2X7	Human Peripheral blood cells	SNP 1068T→C was found positively associated with resistance to both congenital and ocular toxoplasmosis	[118]
*T. gondii*	P2X7	Murine peritoneal cell	In vivo infection of P2X7-deficient mouse resulted in a more severe acute infection, higher parasite burdens and pronounced liver pathology	[119]

Several studies have reported the involvement of purinergic receptors in viral infections, either as facilitators or as host defense factors. Purinergic receptor antagonists, such as suramin, pyridoxal-phosphate-6-azophenyl-2’ ,4’-disulfonate (PPADS) and brilliant blue G (BBG), the latter of which is a selective blocker of P2X7R, have been used to block the infection of hepatocytes by the hepatitis B virus [120-122]. Suramin has been demonstrated to exert antiviral effects on other animal viruses [123,124], although no further studies have associated the antagonistic action of this compound with purinergic receptors. Moreover, human endothelial cells infected in vitro with cytomegalovirus displayed significantly increased expression of P2Y1R, P2Y2R and P2X7R compared to uninfected cells, although only slight effects were found following infection with the herpes virus, likely indicating a virus-specific effect [125].

Despite all of the efforts to control and prevent the spread of human immunodeficiency virus (HIV) type 1 (HIV-1), HIV-1 infection and the resulting acquired immunodeficiency syndrome (AIDS) remain public health problems worldwide. HIV is an enveloped virus classified into a subgroup of retroviruses termed lentiviridae because these viruses exhibit extended clinical latency and persistent viral replication throughout the illness [126-130]. The continuous replication of HIV-1 and chronic immune activation mediate the drastic depletion of CD4+ T cells, a hallmark of infection with HIV-1 and other associated immune disorders [131-134]. Throughout the course of infection, immune responses only partially control the level of these viruses in the blood [135].

The emergence of highly active antiretroviral therapy (HAART) has enabled significant improvements in the management of HIV-infected patients: the reduction of plasma HIV-1 levels below the level of detection of commercially available tests and limited immune reconstitution [136-139]. The use of HAART has resulted in a significant reduction in AIDS-related morbidity and mortality [140]. However, although HAART efficiently delays the onset of AIDS, its clinical utility is limited by several barriers, such as viral resistance, non-adherence to therapy and drug toxicity. Therefore, the search for new targets of HIV-1 and/or host cellular proteins is essential for the success of HIV-1 treatment [141-143].

Three recent reports have directly assessed the possible involvement of purinergic receptors in the immunopathogenesis of HIV-1 infection, raising the possibility of using these host proteins as targets for HIV-1 treatment. Séror et al. found that infecting human cells with HIV-1 leads to the release of ATP via pannexin-1 hemichannels and that this event is essential for the initial infection. The ATP-degrading enzyme apyrase prevents HIV-1 infection. Similarly, pharmacological inhibition of pannexin-1 hemichannels or depletion of this protein using small interfering RNAs protected the targeted cells from HIV-1-mediated cell death and prevented HIV-1 infection. General inhibitors of purinergic receptors blocked the replication of X4-tropic and R5-tropic HIV in activated T lymphoblasts and that of R5-tropic HIV in macrophages and dendritic cells. The selective depletion of mRNAs encoding diverse purinergic receptors using interfering RNAs facilitated the identification of P2Y2R as a receptor related to HIV infection. Immunohistochemistry has revealed elevated levels of P2Y2R in lymphoid tissue, the frontal cortex and circulating leukocytes in untreated carrier patients compared with uninfected patients. Immunofluorescence microscopy has shown that P2Y2R is polarized at the virological synapse. Pharmacological inhibition or genetic ablation of P2Y2R has reproduced the effects of general purinergic receptor blockers. Ultimately, proline-rich tyrosine kinase 2 (Pyk2), a downstream effector of P2Y2R, was found to be a critical mediator of HIV-1 infection [144].

The second study demonstrated that inhibiting the purinergic receptors of macrophages resulted in a significant reduction in the rate of HIV replication. Macrophages are indispensable in HIV pathogenesis because they are susceptible to productive infection, often in the absence of cytopathic or deleterious effects, thus serving as long-lived virus reservoirs [145,146]. Therefore, improving the understanding of the mechanisms by which HIV infects and replicates within macrophages is crucial for designing appropriate strategies to reduce the latency and spread of HIV-1 [147]. Using oxidized ATP, a P2XR antagonist, Hazleton et al. detected significant inhibition of HIV replication in macrophages in a dose-dependent and viral strain-independent manner, indicating that purinergic receptors may be required for HIV replication. To further discriminate which P2Rs may be involved, macrophages were treated with specific pharmacological P2R antagonists. This approach revealed the requirement of at least three purinergic receptors, P2X1R, P2X7R and P2Y1R. Moreover, using a β-lactamase HIV entry assay, P2X1R was demonstrated to participate in the viral entry of macrophages. Finally, treating primary cultures of human macrophages with HIV gp120 resulted in significantly increased release of ATP, indicating the possible autocrine regulation of critical events in the viral life cycle [148].

In the third study, P2XR antagonists inhibited HIV-1 from infecting CD4+ T lymphocytes via both cell-free and cell-to-cell contact in a dose-dependent manner. Additionally, exploration of a library of purinergic antagonists demonstrated that P2XR antagonists are the most potent inhibitors of HIV-1 fusion, providing evidence of a new therapeutic target to prevent HIV-1 infection of CD4+ T lymphocytes [149].

Purinergic receptors have also been associated with the neurotoxicity caused by HIV in infected individuals. In the central nervous system, microglia are activated by HIV-1, and in response, these cells release neuroinflammatory and excitotoxic products that are associated with the pathogenesis of neuroAIDS in many infected individuals [150]. Because P2XRs are key regulators of microglial functions, Sorrel and Hauser investigated whether these receptors are involved in the neurotoxic effects of HIV and morphine in infected individuals, as it has been reported that opioid-dependent microglial activation is mediated by P2X4R signaling. Pretreatment of microglia with TNP-ATP, a nonselective stimulator of P2XRs, inhibited Tat- and/or morphine-related neuronal death in a dose-dependent manner and prevented the increase in cytosolic free Ca^2+^. They also detected a rise in the level of ATP in the medium of neuron-glia co-cultures after 30 minutes of incubation in Tat and morphine, either individually or in combination. Finally, using P2XR antagonists and agonists, P2X4R was identified as the receptor responsible for neurotoxicity in HIV-infected individuals [151]. Similarly, large amounts of ATP, ADP and AMP and small amounts of adenosine and glutamate were detected in the supernatant of HIV-infected macrophage cultures. Applying diluted aliquots of these supernatants to neuronal cultures increased the amount of extracellular glutamate and decreased the neuronal spine density via mechanisms that are dependent on purinergic and glutamatergic receptor activation [150].

Previous investigations have also explored the role of adenosine receptors in the pathogenesis of AIDS. Pingle et al. demonstrated a protective effect of stimulating the A1A receptor (A_1A_R) against HIV-1 Tat-induced toxicity in primary cultures of rat cerebellar granule neurons and in rat pheochromocytoma (PC12) cells. Activation of A_1A_R ameliorated the Tat-mediated changes in PC12 cells, such as the increase in the intracellular Ca^2+^ content, the release of NO and the expression of inducible nitric oxide synthase (iNOS). Furthermore, pretreatment with an A_1A_R agonist reduced the level of activation of NF-κB by Tat and the number of apoptotic cells [152]. Moreover, A_2A_R stimulation inhibited the Tat-induced production of TNF-α, a cytokine that plays a pathological role in HIV-associated dementia, by macrophages [153]. Together, these studies suggest that modulating the activity of the adenosine receptor may be helpful in preventing HIV-1-associated abnormalities [154]. Furthermore, another report showed that stimulating the A2A receptor using a monoclonal antibody reduced the level of expression of the chemokine receptors CXCR4 and CCR5 in CEM T-cells, indicating a putative mechanism that could be exploited to block the entry of HIV-1 [155].

## Conclusion

Purinergic receptors are considered to be powerful modulators of several physiological and pathological events, making them attractive molecular targets for pharmacological research. Although great advances in the management of HIV-infected patients have been achieved due to the emergence of HAART, barriers such as drug resistance, drug toxicity and the cost of treatment necessitate the development of new antivirals [156]. Accordingly, recent reports of the role of purinergic receptors in the immunopathogenesis of HIV-1 infection indicate that they are putative pharmacological targets that should be further explored. In addition, ATP could strengthen the function of the innate and adaptive immune systems because it modulates immunological events that are crucial for the anti-HIV response. For instance, prostaglandin E2 (PGE2) inhibited HIV-1 replication in macrophages via a protein kinase A-dependent mechanism [157]. Interestingly, P2X7R stimulation by ATP is required for the release of PGE2 and other autacoids [158]. Additionally, Leal and colleagues found a significant increase in the expression level of CD39 (NTPDase-1) in lymphocytes from HIV-patients, which correlated to a significant increase in the ATP- and ADP-hydrolytic activities [159]. These findings indicate that extracellular nucleotides might be closely associated with the immune response to HIV infection.

Therefore, further studies must be conducted to improve the understanding of the extent and impact of purinergic signaling activities during all stages of HIV-1 infection.

## First round

## Reviewer’s report

### Reviewer 1: Neil S. Greenspan, Case Western Reserve University, United States of America

### Reviewers’ comments

Pacheco et al. review several aspects of purinergic signaling including the receptors and ligands and their roles in immune responses, bacterial infections, protozoal infections, and HIV infection. A quick survey on PubMed of recent reviews on purinergic signaling reveals none focused precisely on the roles of purinergic ligands and receptors in HIV infection. Thus, the role of purinergic signaling in HIV infection is a topic deserving of review.

The sections on the effects of purinergic signaling in the context of either bacterial or protozoal infections are too brief to be of much use and distract from the main focus on the connections between purinergic signaling and HIV pathogenesis. Similarly, at the beginning of the section pertaining to HIV infection, mentions of effects of purinergic receptor antagonists on infection by “hepatitis virus” (which one, A?, B?, C?, D?) or the effects of cytomegalovirus on expression of purinergic receptors seem gratuitous and can be deleted.

Answer 1:* We did the alterations recommended and focused the text in the HIV-1.*

The focus needs to be more specifically on how purinergic ligands and receptors participate in HIV pathogenesis. Greater integration of the material addressing effects of purinergic molecules on immune responses in general and on HIV infection in particular would strengthen the article. The authors would also add value to their review by identifying specific experimental questions deserving further exploration.

Answer 2:* We did the alterations recommended.*

Quality of written English: Not suitable for publication unless extensively edited.

Answer 3:* We sent the paper to American Journal Experts. The certificate is attached.*

## Reviewer’s report

### Reviewer 2: Rachel Gerstein, University of Massachusetts Medical School, United States of America

Report form:

Overall, this review needs considerable revision to be more effective and readable.

Some specific comments that might guide the revision –

Abstract: An abstract should stand alone, and not require specialized knowledge to be understood by the reading audience. And most important, it must give a good idea of what the article is about. To state that there is a “relationship between […] purinergic signaling […] and immunopathogenesis of HIV-1” is very vague.

Answer 1:* We did the alterations recommended.*

Intro and body of review:

1. Is purinergic signaling important in immune **responses** ? is ATP a DAMP for mature functional immune cells ? I was not convinced.

Answer 2:* We did the alterations recommended.*

3. The section on thymocytes does not seem relevant to a discussion of response to a virus.

Answer 3:* In the process of HIV-1 infection, one of the consequences is the reduction of the timopoiesis. The maturation and differentiation of thymocytes are modulated by IL-7 / IL-7 receptor (IL-7R) signaling pathway. During infection by HIV-1 occurs reduction in the level of IL-7 (Young and Angel, 2011).*

*Young CD*^*1*^*, Angel JB. HIV infection of thymocytes inhibits IL-7 activity without altering CD127 expression. Retrovirology. 2011 Sep 16;8:72. doi: 10.1186/1742-4690-8-72.*

4. The mention of TH17 profile, the context (ie “this condition”) is unclear.

Answer 4:* We changed this in the text.*

5. If the authors want to highlight work most related to HIV-1, the inclusion of the sections on bacterial and protozoan infections are not needed.

Answer 5:* We changed this in the text.*

6. In the last part of section 4, in the “neuroAIDS” section, citations are needed to document the connection and data indicating that ATP levels rise in the brain and that this is tied to cognitive impairment.

Answer 5:* We added the reference below in the text.*

Luis B. Tovar-y-Romo & Dennis L. Kolson & Veera Venkata Ratnam Bandaru & Julia L. Drewes & David R. Graham & Norman J. Haughey. Adenosine Triphosphate Released from HIV-Infected Macrophages Regulates Glutamatergic Tone and Dendritic Spine Density on Neurons. J Neuroimmune Pharmacol. DOI 10.1007/s11481-013-9471-7.

7. Recent papers should be mentioned:

PMID:24842759, PMID:24158495 and especially: Idzko M, Ferrari D, Eltzschig HK., Nature. 2014 May 15;509(7500):310–7. doi: 10.1038/nature13085. PMID:24828189

Answer 6:* We commented and added the reference above in the text.*

Quality of written English: Not suitable for publication unless extensively edited.

Answer 7:* We sent the paper to American Journal Experts. The certificate is attached.*

## Second round

## Reviewer’s report

### Reviewer 1: Neil S. Greenspan, CaseWestern Reserve University, United States of America

Comments to Authors:

Pacheco et al. have responded substantively to the previous reviews by tightening the focus of the manuscript to emphasize the role of purinergic receptors in the pathogenesis of HIV-1 infection of humans. The writing has also been substantially improved, although there are still a small number of passages requiring revision. The following comments note areas of the text requiring author responses.

1. In the abstract, the last two sentences are in need of editing.

“Three recent reports have examined the relationship between the level of extracellular ATP, the mechanisms underlying purinergic receptors participating of in the infection mechanism of HIV-1 in the cell. Although preliminary, these results indicate that purinergic receptors are putative pharmacological targets to that should be further explored in future studies.”

2. The second-to-last sentence at the end of the first paragraph continuing onto the top of page 4 appears to make more sense if “Although” and “it” are deleted. “Although The detailed signaling process that is triggered by the activation of most P2XRs, it has yet to be completely elucidated.”

3. On the top of page 5, in the sentence beginning “A large body of evidence …,” the word “responses” might better be replaced by “phenomena,” as immune “responses” are most typically referred to by immunologists to indicate specifically coordinated events involving the proliferation and differentiation of lymphocytes and not separately for every sort of molecular or cellular mechanism that arises in the course of an immune response.

4. The mediator adenosine is referred to as a “danger” molecule. While I recognize that this usage is widespread, I would advise not using it especially when, as here, “danger” is not defined. Frequently, molecules that are taken to correspond to “danger signals,” with minimal to no justification are in fact, in some circumstances, causes of danger. For example, I just saw a report that the massive tissue damage that characterizes Ebola virus infection is largely attributable not to direct virus-mediated effects but to the host response, in particular so-called cytokine storm (http://www.npr.org/blogs/goatsandsoda/2014/08/26/342451672/howebola-kills-you-its-not-the-virus) from my perspective, calling the release of adenosine a danger signal adds no insight not gleaned from a careful delineation of the effects of adenosine on various receptors and signaling pathways and can easily lead to incorrect conclusions and sloppy thinking.

5. On page 9, in the first full paragraph, it is stated that about HIV-1 infection that: “viruses in the blood, which remains measurable throughout the course of infection …” The next paragraph seemingly contradicts that assertion (“…the reduction of plasma HIV-1 levels below the level of detection of commercially available tests and limited immune reconstitution (139–142).”).

6. On page 13, I would add one word to the sentence quoted below, “Accordingly, recent reports of the role of purinergic receptors in the immunopathogenesis of HIV-1 infection indicate that they are putative pharmacological targets that should be further explored”.

Quality of written English: Acceptable

### Authors’ response

We accept all of the reviewer’s suggestions. The text has been changed to accommodate them.

### Reviewer’s report by Rachel Gerstein

University of Massachusetts Medical School, United States of America.

This reviewer provided no comments for publication.

## Competing interests

The authors declare that they have no competing interests.

## Authors’ contributions

PAFP wrote the main body of the review and LGBF reviewed and formatted the structure of the text. RXF has reviewed the topics about purinergic receptor and ICNPP has reviewed topics related to HIV-1 infection. All authors read and approved the final manuscript.
